# Aging Reduces the Activation of the mTORC1 Pathway after Resistance Exercise and Protein Intake in Human Skeletal Muscle: Potential Role of REDD1 and Impaired Anabolic Sensitivity

**DOI:** 10.3390/nu8010047

**Published:** 2016-01-15

**Authors:** Marc Francaux, Bénédicte Demeulder, Damien Naslain, Raphael Fortin, Olivier Lutz, Gilles Caty, Louise Deldicque

**Affiliations:** Institute of Neuroscience, Université catholique de Louvain, Louvain-la-Neuve 1348, Belgium; benedicte.demeulder@gmail.com (B.D.); damien.naslain@uclouvain.be (D.N.); raphael-fortin@hotmail.com (R.F.); olivierlutz@msn.com (O.L.); gilles.caty@uclouvain.be (G.C.); louise.deldicque@uclouvain.be (L.D.)

**Keywords:** anabolic resistance, sarcopenia, protein synthesis, protein degradation, autophagy

## Abstract

This study was designed to better understand the molecular mechanisms involved in the anabolic resistance observed in elderly people. Nine young (22 ± 0.1 years) and 10 older (69 ± 1.7 years) volunteers performed a one-leg extension exercise consisting of 10 × 10 repetitions at 70% of their 3-RM, immediately after which they ingested 30 g of whey protein. Muscle biopsies were taken from the vastus lateralis at rest in the fasted state and 30 min after protein ingestion in the non-exercised (Pro) and exercised (Pro+ex) legs. Plasma insulin levels were determined at the same time points. No age difference was measured in fasting insulin levels but the older subjects had a 50% higher concentration than the young subjects in the fed state (*p* < 0.05). While no difference was observed in the fasted state, in response to exercise and protein ingestion, the phosphorylation state of PKB (*p* < 0.05 in Pro and Pro+ex) and S6K1 (*p* = 0.059 in Pro; *p* = 0.066 in Pro+ex) was lower in the older subjects compared with the young subjects. After Pro+ex, REDD1 expression tended to be higher (*p* = 0.087) in the older group while AMPK phosphorylation was not modified by any condition. In conclusion, we show that the activation of the mTORC1 pathway is reduced in skeletal muscle of older subjects after resistance exercise and protein ingestion compared with young subjects, which could be partially due to an increased expression of REDD1 and an impaired anabolic sensitivity.

## 1. Introduction

Sarcopenia has been defined as the decline in skeletal muscle mass and strength with advancing age [1]. The impact of sarcopenia on older people is far reaching as it leads to frailty, loss of mobility, increased risk of cardiovascular and metabolic diseases and mortality [2]. As a result, sarcopenia presents a significant socio-economic load. To date, effective strategies to escape from or at least to attenuate sarcopenia remain limited. Resistance exercise and adequate protein-based nutrition are the current most effective means to limit the loss of muscle mass with aging [3]. However, the benefits of exercise and nutritional interventions to counteract sarcopenia may be partially reduced by a blunted responsiveness to these stimuli [4,5]. This phenomenon has been termed “anabolic resistance” to reflect the inability of the muscle to maintain its protein mass by appropriate stimulation of protein synthesis, a major mechanism contributing to sarcopenia [6]. Periods of disuse or unloading have been shown to exacerbate muscle anabolic resistance [7]. Although growing evidence indicates that anabolic resistance to protein-rich nutrition and resistance exercise probably contributes to the loss of muscle mass during aging, it is currently unclear whether alterations in basal, fasted protein metabolism also play a role [3].

At a cellular level, muscle atrophy originates from prolonged periods of negative net protein balance, which can be due to dampened rates of muscle protein synthesis, elevated rates of muscle protein breakdown, or a combination of both. Measuring the rate of protein breakdown is methodologically less straightforward than the rate of protein synthesis and therefore less conclusive [8]. Therefore, the regulation of protein synthesis after resistance exercise and/or amino acids ingestion in the elderly is far more documented than the regulation of protein breakdown. Recent work indicates that the elderly have a blunted muscle protein synthetic response to amino acid administration [9,10,11] and physical activity [4,12] when compared with that in the young.

At a molecular level, the mammalian target of rapamycin complex 1 (mTORC1) is an essential site of integration for anabolic signals, such as amino acids, insulin, and resistance exercise, to stimulate muscle protein synthesis in human skeletal muscle via, amongst other downstream targets, ribosomal protein S6 kinase 1 (S6K1) and eukaryotic initiation factor 4E-binding protein 1 (4E-BP1) [13]. Basal total protein levels of mTOR, S6K1, and 4E-BP1 have been found to be down regulated in old compared with young individuals [11]. According to the authors, differences in the availability of such key regulatory proteins may contribute to the reduced capacity of the muscle protein synthetic machinery to sense a nutrient signal in aging muscle. In addition essential amino acids-induced mTOR, S6K1, and 4E-BP1 phosphorylation was reduced in old individuals [11]. S6K1 phosphorylation was also impaired after intravenous insulin and amino acid infusions in skeletal muscle of old compared with young individuals but mTOR and 4E-BP1 phosphorylations increased similarly in both groups [14]. Not only the response to amino acids and insulin can be impaired in the elderly, the response to resistance exercise on mTOR, S6K1, and 4E-BP1 phosphorylations has been found to be altered both in the fasted state [4,12] and after amino acids ingestion [15]. Both AMP activated protein kinase (AMPK) [16] and regulated in development and DNA damage responses (REDD1) [17] have been suggested to contribute to the impairment of the mTOR pathway after resistance exercise in the elderly. On the other hand, equivalent response to amino acid ingestion [18] and resistance exercise [19,20] on the mTORC1 pathway between young and old subjects has been found as well, underlying the fact that anabolic resistance is not systematically present in the elderly and that healthy active people are probably less prone to sarcopenia.

As mentioned above the effect of aging on the regulation of protein breakdown in human skeletal muscle has been less studied. Muscle specific RING finger-1 (MuRF-1) and the transcription factor forkhead box (Foxo) at rest and muscle atrophy F-box muscle (MafBx) after resistance exercise were found to be more expressed at the mRNA level in old than young skeletal muscle [21], which was not confirmed by two other studies [17,22]. In one of the latter study, two key makers for autophagy, the microtubule-associated protein 1 light chain 3 (LC3b) I/II ratio and gamma-aminobutyric acid receptor-associated protein (GABARAP), were decreased after resistance exercise, but no difference was found between old and young subjects [22]. Interestingly, the expressions of beclin-1 and autophagy-related protein 7 (Atg7) were consistently increased in old *vs.* young individuals, which could indicate a greater autophagic capacity in the elderly [22].

The present study was carried out given the importance of having a clear picture of both protein synthesis and protein breakdown when trying to understand the mechanisms regulating anabolic resistance and the loss of muscle mass with aging and given the lack of study having analyzed both processes systematically at the same time. Based on previous reports we hypothesized that the activation of the mTORC1 pathway would be attenuated in old individuals and that markers for the ubiquitin-proteasome system as well as for autophagy would be activated more in the elderly compared with young individuals after protein intake and resistance exercise, resulting in a less favorable environment for muscle mass accretion.

## 2. Materials and Methods

### 2.1. Subjects

Nine young and 10 older subjects participated in the present study. The characteristics of the subjects are presented in Table 1. Each subject underwent a medical screening consisting of an electrocardiogram, blood pressure measurement, urine analysis, and blood analysis. Subjects under statin, corticoid treatment, or chemotherapy were excluded. Subjects were physically active but not engaged in a specific strength training program. They received a written and oral report of the study and signed an informed consent. The study was approved by the Medical Ethic Committee of the Université catholique de Louvain, and the investigation was performed according to the principles outlined in the declaration of Helsinki.

**Table 1:** Subjects characteristics.

	Young	Older
Number (n)	9 M	6 M + 4 F
Age (years)	22 ± 0.1	69 ± 1.7 ***
Weight (kg)	78 ± 2.0	79 ± 3.5
Height (cm)	177 ± 2.5	174 ± 3.2
BMI (kg/m^2^)	25 ± 1.0	26 ± 0.9
Total load lifted (kg)	2271 ± 51.4	M + W: 1194 ± 86.3 *** M only: 1365 ± 84.3 *** W only: 981 ± 75.0 ***^, #^

All values are means ± SEM. *** *p* < 0.001 older *vs.* young; # *p* < 0.05 W *vs.* M. M, men; W, women.

### 2.2. Protocol

**Figure 1:**
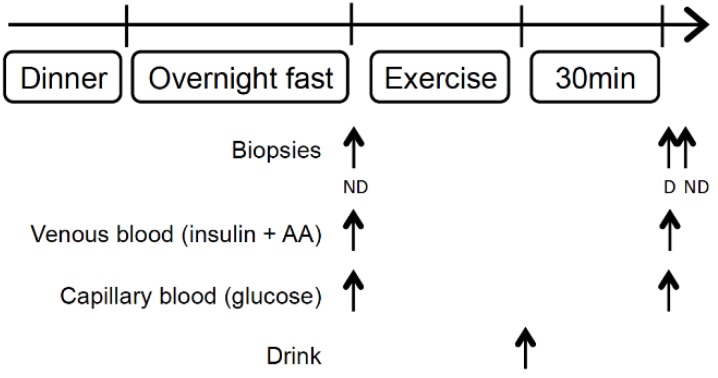
Schematic study protocol. D, dominant; ND, nondominant; AA, amino acids. The exercise consisted in single leg extension of the dominant leg.

The subjects were asked to refrain from heavy or vigorous physical activity 48 h prior to the experimental sessions. The subjects came twice to the laboratory. During the first session, the 3-repetition maximum (3-RM) of the dominant leg was determined on a leg extension machine. The exercise consisted of knee extensions movements from 90° to 160° with increasing load until only 3 repetitions could be performed with 3 min rest between the different sets. The day of the second session, the subjects arrived at 6 a.m. at the laboratory after a standardized dinner the prior evening (63% carbohydrates, 13% proteins, and 24% fats) and a 10-h overnight fast. After a 30-min rest, the first biopsy was taken in the non-dominant leg (Fasted) with a 4-mm Bergström biopsy needle in the mid portion of the vastus lateralis after local anesthesia (1% lidocaine). They then performed 10 sets of 10 leg extensions at 70% of their 3-RM with their dominant leg with 3 min rest between each set. Young and older subjects differed in the total load lifted defined as the weight lifted multiplied by the number of repetitions effectively performed. The total load lifted by the young subjects was almost twice the load lifted by the older subjects (*p* < 0.001, Table 1). Of note, a gender effect was measured in the load lifted in the older group (*p* < 0.05) while no other difference was observed between older men and women for any molecular marker measured. All results are therefore presented for the whole older group. Immediately at the end of the exercise bout, they ingested a drink rich in proteins (30 g of whey protein hydrolysate, of which 2.5 g was leucine). Thirty minutes after, a second muscle biopsy was taken in the exercised (dominant) leg (Pro+ex) and a third biopsy in the non-dominant leg (Pro). The two biopsies in the non-dominant leg were performed in the same incision, the first one with the needle pointing proximally and the second one with the needle pointing distally to reduce potential activation of inflammatory signaling pathways, as recommended by Van Thienen *et al.* [23]. Blood, fat, and connective tissue were removed immediately and the sample was frozen in liquid nitrogen and stored at −80 °C until further analyses. Venous blood samples were taken at rest and 30 min after the ingestion of the drink in heparinized tubes (Figure 1). At the same time, blood glucose was determined from a fingertip puncture using the Glucocard X-Meter (Arkray, Kyoto, Japan).

### 2.3. Blood Chemistry

Blood samples were centrifugated at 3000 *g* for 6 min and plasma was stored at −20 °C. Plasma insulin concentration was determined by ELISA using the ultrasensitive human insulin kit from Mercodia (Uppsala, Sweden), following the manufacturer instructions. Plasma aminogram was determined using the Amino Acid Analyser Biochrom 30 (Isogen Life Sciences, Temse, Belgium).

### 2.4. Western Blotting

Frozen muscle samples were homogenized in a buffer containing 20 mM Tris, pH 7.0, 270 mM sucrose, 5 mM EGTA, 1 mM EDTA, 1% Triton X-100, 1 mM sodium orthovanadate, 50 mM sodium β-glycerophosphate, 5 mM sodium pyrophosphate, 50 mM fluoride, 1 mM DTT (1,4-dithiothreitol), and a protease inhibitor cocktail containing 1 mM EDTA (Roche Applied Science, Vilvoorde, Belgium). After centrifugation at 4 °C at 10,000 *g* for 15 min, the supernatants were stored at −80 °C. Protein concentration was determined using the DC protein assay kit (Biorad, Nazareth Eke, Belgium) with bovine serum albumin as a standard. For immunoblotting, 30 µg of proteins were subjected to sodium dodecyl sulfate polyacrylamide gel electrophoresis (SDS-PAGE) and transferred onto polyvinlidene difluoride (PVDF) membrane. After a blocking step of 1 h, membranes were probed with the following primary antibodies overnight: phospho-Thr389 S6K1, phospho-Ser2448 mTOR, phospho-Thr246 proline-rich Akt substrate of 40 kDa (PRAS40), phospho-Ser473 Akt/protein kinase B (Akt/PKB), phospho-Thr308 Akt/PKB, phospho-Thr172 AMPK, phospho-Ser79 Acetyl CoA carboxylase (ACC), LC3b, hypoxia-inducible factor 1 alpha (HIF-1α), eukaryotic initiation factor 2 (eEF2) (Cell Signaling, Leiden, The Netherlands), p62 (Progen, Heidelberg, Germany), and REDD1 (ProteinTech, Manchester, UK). The appropriate secondary antibody conjugated to peroxidase and the BM chemiluminescence blotting system (Roche, Meylan, France) were used for detection with the GBox system using the softwares GeneSnap to capture the images and GeneTools to quantify the bands (Syngene, Cambridge, UK). Preliminary experiments showed that total eEF2 was stable throughout the experimental conditions and could therefore be used as a loading control. On a subset of samples, we also found that the total forms of the proteins were not modified by any condition. After correction for the inter-membranes variation using an internal standard, the results are presented as the ratio (phospho-) protein of interest/eEF2. The internal standard consisted of the same sample loaded on each gel to which an arbitrary value of 100 was given during the quantification step of each membrane and to which all other samples were reported.

### 2.5. Real-Time Quantitative Polymerase Chain Reaction

Muscles samples (~20 mg) were homogenized with the TissueLyser II (Qiagen) in 1 mL Trizol^®^ reagent (Invitrogen, Merelbeke, Belgium). RNA isolation was performed according to the manufacturer’s instructions. RNA quality and quantity were assessed by Nanodrop^®^ spectrophotometry (Thermofisher, Gent, Belgium). Reverse transcription was achieved from 1 µg RNA using the iScriptTMcDNA Synthesis Kit from Bio-Rad (Nazareth, Belgium), according to the manufacturer’s instructions. Primers used for real-time quantitative PCR are listed in Table 2. Experiments were performed on a MyIQ2 thermocycler (Bio-Rad), with the following conditions: 3 min at 95 °C, followed by 35 cycles of 30 s at 95 °C, 30 s at 60 °C, and 30 s at 72 °C. For each gene, all samples were run in duplicate on the same plate. 10 µL volume containing 4.8 µL IQ SybrGreen SuperMix (Bio-rad), 0.1 µL of each primer (100 nM final) and 5 µL cDNA at the appropriate dilution was used for each reaction. Quality control was realized by the assessment of the melting curve. To compensate for variations in input RNA amounts and efficiency of reverse transcription, cyclophilin A (Cyclo A) and ribosomal protein L 4 (RPL4) mRNA were quantified, and results were normalized to these values. These genes were chosen out of four normalization genes (Cyclo A, RPL4, beta-2-microglobulin and glyceraldehyde 3-phosphate dehydrogenase) using the GeNorm applet according to the guidelines and theoretical framework described elsewhere [24].

**Table 2:** Primers sequences.

	Forward	Reverse
BNIP3	CTG AAA CAG ATA CCC ATA GCA TT	CCG ACT TGA CCA ATC CCA
BNIP3L	CCA AGG AGT TCC ACT TCA GAC	AGT AGG TGC TGG CAG AGG GTG T
CycloA	CTT CAT CCT AAA GCA TAC GGG TC	TGC CAT CCA ACC ACT CAG TCT
GABARAP	GTG CCC TCT GAC CTT ACT GTT G	CAT TTC CCA TAG ACA CTC TCA TC
IGF-I	TAT TTC AAC AAG CCC ACA GG	CAT CTC CAG CCT CCT TAG AT
IGF-II	TGG ACA CCC TCC AGT TC	GGA AAC AGC ACT CCT CAAC
HIF-1α	GCC CCA GAT TCA GGA TCA GA	TGG GAC TAT TAG GCT CAG GTG AAC
LC3b	AAT CCC GGT GAT AAT AGA ACG A	GGA GAC GCT GAC CAT GCT GT
MafBx	CGA CCT CAG CAG TTA CTG CAA C	TTT GCT ATC AGC TCC AAC AG
MuRF-1	AAA CAG GAG TGC TCC AGT CGG	CGC CAC CAG CAT GGA GAT ACA
p62	CCT CTG GGC ATT GAA GTT G	TAT CCG ACT CCA TCT GTT CCTC
RPL4	ATA CGC CAT CTG TTC TGC CCT	GCT TCC TTG GTC TTC TTG TAG CCT

BNIP3, BCL2/adenovirus E1B 19kDa interacting protein 3; BNIP3L, BCL2/adenovirus E1B 19 kDa interacting protein 3-like; CycloA, cyclophilin A; GABARAP; Gamma-aminobutyric acid receptor-associated protein; IGF-I; insulin-like growth factor-1; IGF-II; insulin-like growth factor-2; HIF-1α; hypoxia-inducible factor-1 alpha; LC3b; Microtubule-associated protein 1 light chain 3; MafBx, Muscle Atrophy F-Box Muscle; MuRF-1, Muscle specific RING Finger 1; p62, p62 protein; RPL4, ribosomal protein L4.

### 2.6. Statistical Analysis

All values are expressed as the means ± the standard error of the mean (SEM). A mixed ANOVA model was used with the subjects as a random variable and groups (Young, Old) and conditions (Fasted, Pro, Pro+ex) as fixed independent variables. When appropriate, the Fisher’s LSD test was used to compare means. Statistical significance was set at *p* < 0.05.

## 3. Results

### 3.1. Blood Parameters

The protein-rich drink and exercise increased blood glucose (*p* < 0.001) and plasma insulin (*p* < 0.01), leucine (*p* < 0.001), isoleucine (*p* < 0.001), and valine (*p* < 0.001) concentrations in both young and old subjects compared to the fasted conditions (Table 3). The older subjects had higher glucose levels than the young ones in both Fasted (*p* < 0.05) and Pro+ex (*p* < 0.001) conditions. Insulin increased about four times between Fasted and Pro+ex in the older compared with 2.5 times in the young subjects, resulting in a higher plasma insulin concentration in the older compared with the young group (*p* < 0.05). The opposite effect was measured for isoleucine, which increased less in the older compared with the young group in response to proteins and exercise (*p* < 0.05).

**Table 3:** Plasma glucose, insulin, and branched-chain amino acids concentrations.

	Young		Older	
	Fasted	Pro+ex	Fasted	Pro+ex
Glucose (mg/dL)	90.2 ± 1.33	100.3 ± 2.21 ^§§§^	97.5 ± 2.65 *	111.8 ± 2.43 ^§§§^^,^***
Insulin (µU/mL)	10.3 ± 2.93	26.2 ± 4.72 ^§§^	9.5 ± 1.17	39.7 ± 6.51 ^§§^^,^*
Isoleucine (µmol/L)	87 ± 7.1	164 ± 9.5 ^§§§^	78 ± 5.6	141 ± 9.0 ^§§§^^,^*
Leucine (µmol/L)	171 ± 7.6	408 ± 28.2 ^§§§^	155 ± 10.2	353 ± 24.7 ^§§§^
Valine (µmol/L)	285 ± 14.0	358 ± 18.8 ^§§§^	280 ± 15.2	336 ± 15.9 ^§§§^

All values are means ± SEM. ^§§^ *p* < 0.01, ^§§§^ *p* < 0.001 *vs.* Fasted same age. * *p* < 0.05, *** *p* < 0.001 *vs.* Young same condition.

### 3.2. PKB Response to Exercise and Feeding is Blunted in Old Subjects

**Figure 2:**
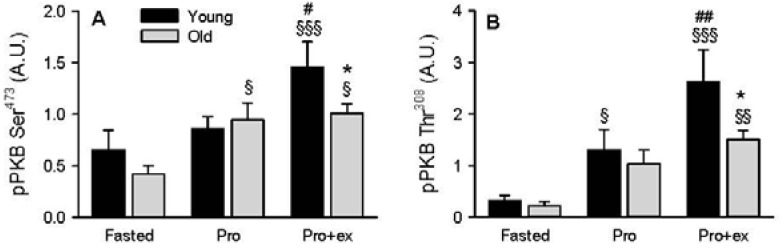

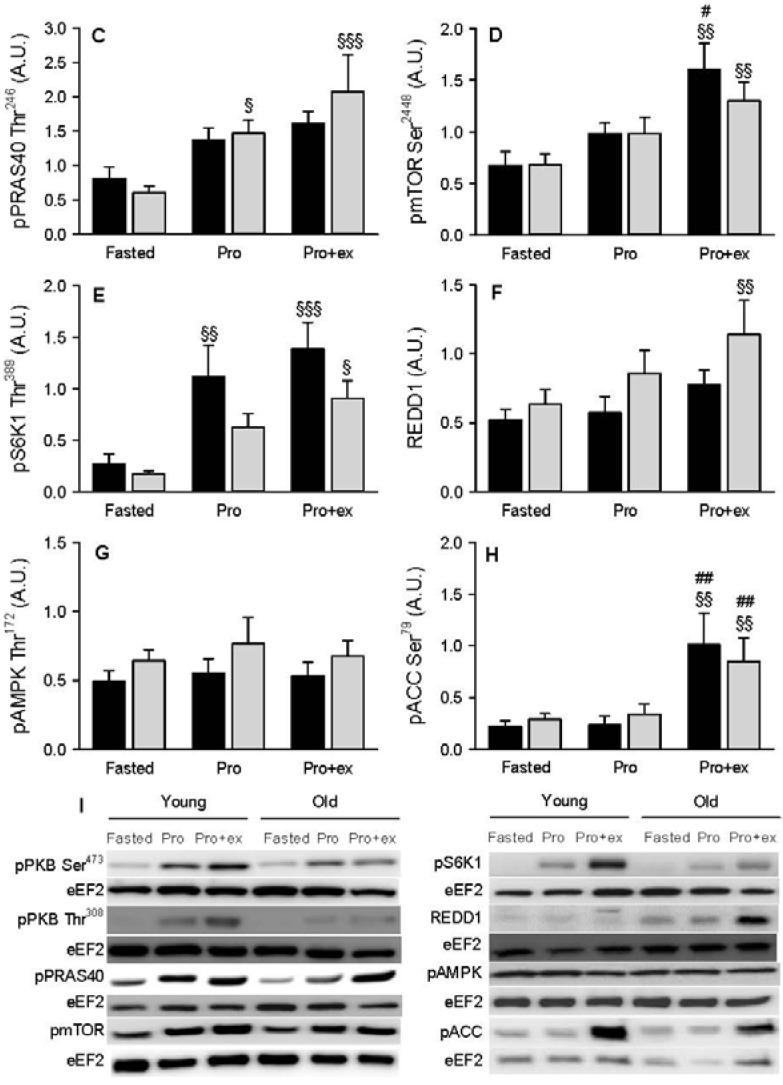
Effect of aging on the PKB/mTOR pathway activation after resistance exercise and protein intake. (**A**) Phospho-PKB Ser^473^; (**B**) phospho-PKB Thr^308^ (**C**) phospho-PRAS40 Thr^246^; (**D**) phospho-mTOR Ser^2448^; (**E**) phospho-S6K1 Thr^389^; (**F**) REDD1 expression; (**G**) phospho-AMPK Thr^172^ and (**H**) phospho-ACC Ser^79^ in Fasted, Pro, and Pro+ex conditions in young and older subjects; (**I**) Representative western blots. Values are means ± SEM. ^§^ *p* < 0.05, ^§§^ *p* < 0.01, ^§§§^ *p* < 0.001 *vs.* Fasted same age. ^#^ *p* < 0.05, ^##^ *p* < 0.01 *vs.* Pro same age. * *p* < 0.05 *vs.* Young same condition.

No difference between young and older subjects was observed on the components and regulators of the PKB/mTORC1 pathway at rest in the fasted state (Figure 2A–H). The phosphorylation of PKB was increased after the protein-rich drink (Ser^473^ *p* < 0.05 in older and Thr^308^ *p* < 0.05 in young group, Figure 2A). Combining protein intake with exercise further increased PKB phosphorylation in the young (*p* < 0.05) but not in the older subjects, resulting in a lower phosphorylation of PKB at Ser^473^ (−30%) and Thr^308^ (−40%) in the older group compared with the young group (*p* < 0.05, Figure 2A,B). Compared with the fasted condition, phospho-PRAS40, a downstream target of PKB [25], increased by two-fold in Pro (*p* < 0.05) and three-fold in Pro+ex (*p* < 0.001) in the older, but not in the young group, (Figure 2C). Another downstream indirect target of PKB is mTOR at Ser^2448^ [26], the latter being up regulated in Pro+ex in both groups compared with the fasted conditions (*p* < 0.01, Figure 2D). Phospho-S6K1 at Thr^389^ is a marker for mTORC1 activity [27] and is known to be highly responsive to amino acids [28]. In all conditions, S6K1 Thr^389^ was always less phosphorylated in the older group (ANOVA, between groups: *p* < 0.05, Figure 2E). In the young group, S6K1 Thr^389^ increased in Pro (four fold, *p* < 0.01) and in Pro+ex (five fold, *p* < 0.001) while in the old group, it only increased in Pro+ex (five fold, *p* < 0.05) (Figure 2E). A trend to a lower phosphorylation of S6K1 at Thr^389^ in the old subjects compared with the young subjects was measured in Pro (*p* = 0.059) and in Pro+ex (*p* = 0.066). REDD1 (Figure 2F) and AMPK (Figure 2G) are potential inhibitors of the PKB/mTOR pathway [29]. While phospho-AMPK Thr^172^ was not modified by any condition, REDD1 expression was higher in elderly (ANOVA, between groups: *p* < 0.05, Figure 2F). It increased by almost two-fold in Pro+ex compared with the fasted conditions in the older subjects only (*p* < 0.01, Figure 2F), resulting in a trend to a higher expression in old subjects compared with young subjects in this condition (*p* = 0.087). Finally, phospho-ACC Ser^79^ was measured as it is a direct target of AMPK and therefore reflects the activity of the latter [30]. Phospho-ACC increased by about four-fold in the young group (*p* < 0.01) and three-fold in the older group (*p* < 0.01) in Pro+ex compared with both Fasted and Pro, without any difference between both groups (Figure 2H).

### 3.3. Markers for Autophagy and the Ubiquitin-Proteasome System Are Not Altered with Age

**Figure 3:**
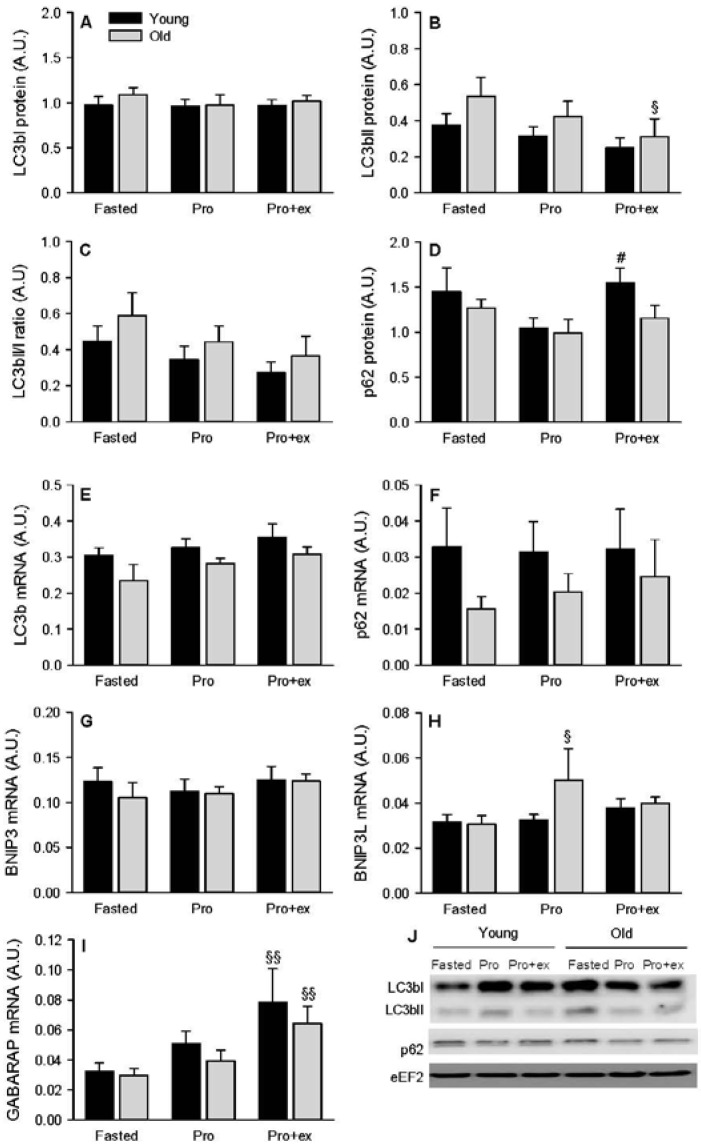
Effect of aging on autophagy markers after resistance exercise and protein intake. (**A**) LC3bI; (**B**) LC3bII; (**C**) LC3bI/LC3bII; (**D**) p62 protein expressions and (**E**) LC3b; (**F**) p62; (**G**) BNIP3; (**H**) BNIP3L; (**I**) GABARAP mRNA levels in Fasted, Pro, and Pro+ex conditions in young and older subjects; (**J**) Representative western blots. Values are means ± SEM. ^§^ *p* < 0.05, ^§§^ *p* < 0.01 *vs.* Fasted same age. ^#^ *p* < 0.05 *vs.* Pro same age.

The autophagic flux was assessed by measuring LC3b and p62 protein expressions. An increase in the flux is featured by increased LC3bII protein level and LC3bII/I ratio associated to a decreased p62 protein content [31]. We therefore quantified those protein expressions and calculated the ratio LC3bII/LC3bI. While LC3bI expression was unmodified by any condition (Figure 3A), LC3bII tended to globally decrease in Pro and Pro+ex compared with the fasted conditions with a significant difference between Pro+ex *vs.* Fasted in the older group (*p* < 0.05, Figure 3B). The ratio LC3bII/I tended to follow the same pattern of regulation as LC3bII, and no difference was observed in any condition (Figure 3C). In the young group, p62 expression was higher in Pro+ex compared with Pro alone (*p* < 0.05, Figure 3D). Altogether, those results showed no activation of autophagy. Nevertheless, the autophagic program can also be regulated transcriptionally by increasing the expression of genes involved in the regulation of autophagy itself [32]. LC3b mRNA was systematically lower in the older group (ANOVA, between groups: *p* < 0.05, Figure 3E). LC3b (Figure 3E), p62 (Figure 3F), and BNIP3 (Figure 3G) mRNA levels were not modified by any condition. BNIP3L mRNA increased in Pro compared with Fasted in the old subjects only (+60%, *p* < 0.05), which resulted in a trend to higher values in the older subjects compared with the young subjects (*p* = 0.064, Figure 3H). The last marker analyzed for the activation of the transcriptional program of autophagy, GABARAP, increased in Pro+ex in both young (+140%) and older group (+120%, *p* < 0.01, Figure 3I).

**Figure 4:**
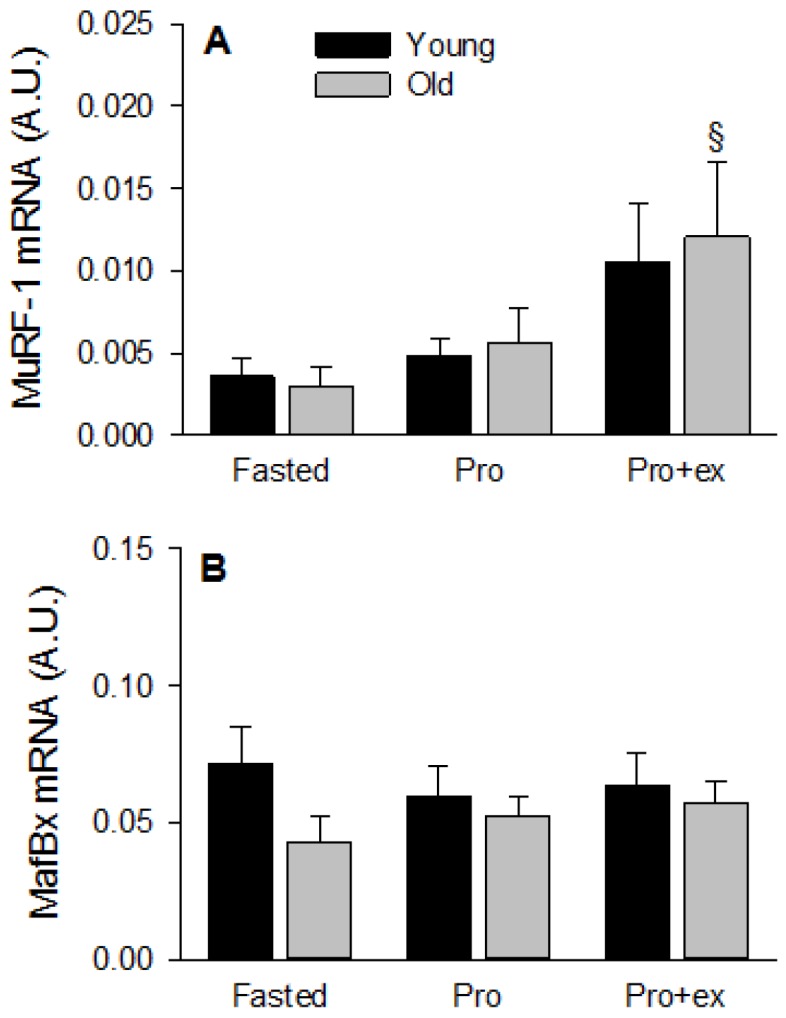
Effect of aging on markers for the proteasome-ubiquitin system after resistance exercise and protein intake. (**A**) MuRF-1 and (**B**) MafBx mRNA levels in Fasted, Pro, and Pro+ex conditions in young and older subjects. Values are means ± SEM. ^§^ *p* < 0.05 *vs.* fasted same age.

In addition to autophagy, the ubiquitin-proteasome pathway is another key proteolytic system in skeletal muscle. MuRF-1 and MafBx expressions are often quantified as markers for the activation of this system [33]. MuRF-1 mRNA levels increased by three-fold in Pro+ex compared to Fasted conditions and two-fold compared to Pro conditions in both young and older subjects (*p* < 0.05, Figure 4A). MafBx mRNA was not modified by any condition although a trend to lower fasted values was measured in the older group compared with the young group (*p* = 0.060, Figure 4B).

### 3.4. Basal Insulin-Like Growth Factor-1 and Hypoxia-Inducible Factor 1 Alpha MRNA LEVELS Are Reduced in Old Subjects

**Figure 5:**
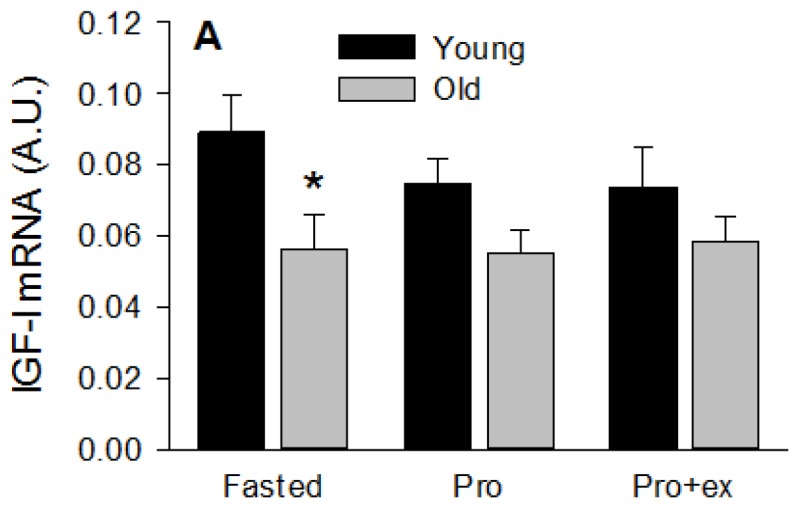

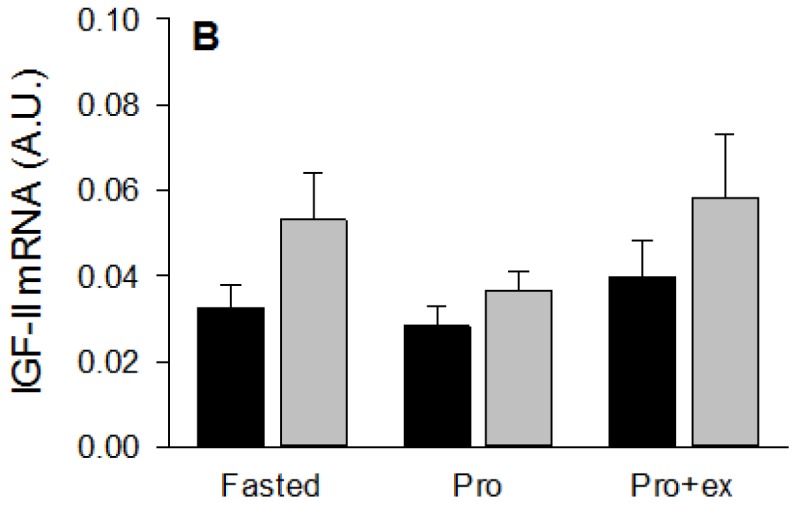
Effect of aging on IGF-I and IGF-II mRNA expression after resistance exercise and protein intake. (**A**) IGF-I and (**B**) IGF-II mRNA levels in Fasted, Pro, and Pro+ex conditions in young and older subjects. Values are means ± SEM. * *p* < 0.05 *vs.* Young same condition.

**Figure 6:**
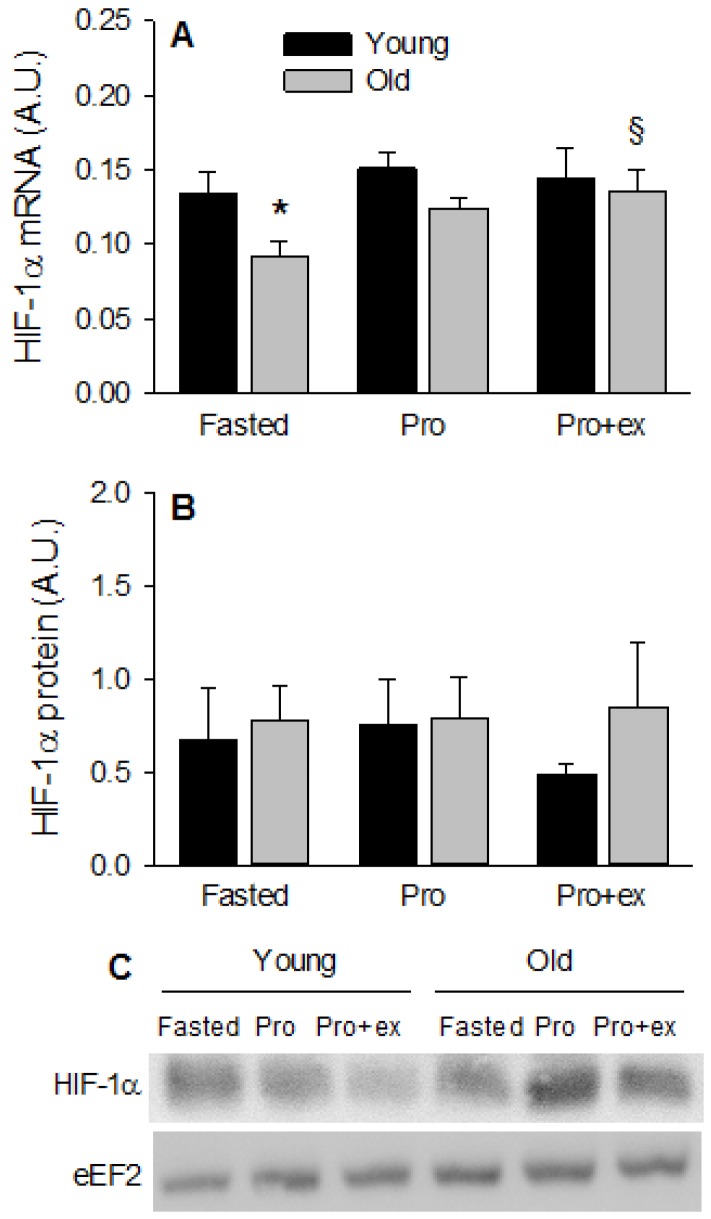
Effect of aging on HIF-1α mRNA and protein expression after resistance exercise and protein intake. (**A**) HIF-1α mRNA and (**B**) HIF-1α protein in Fasted, Pro, and Pro+ex conditions in young and older subjects; (**C**) Representative western blots. Values are means ± SEM. ^§^ *p* < 0.05 *vs.* Fasted same age. * *p* < 0.05 *vs.* Young same condition.

Compared with young subjects, insulin-like growth factor-1 (IGF-I) mRNA level was about 40% less in old subjects at basal (*p* < 0.05, Figure 5A) and remained lower after Prot and Prot+ex (ANOVA, between groups: *p* < 0.05). Inversely, IGF-II mRNA was higher is older subjects (ANOVA, between groups: *p* < 0.05 Figure 5B), and was not modified by any treatment. As hypoxia has been proposed to be a potential contributing mechanism to sarcopenia [34], HIF-1α was quantified at the mRNA (Figure 6A) and protein (Figure 6B) levels. Compared with young subjects, basal HIF-1α mRNA level was about 40% less in older subjects (*p* < 0.05). This difference due to age was not more present in Pro and Pro+ex as HIF-1α mRNA increased by about 35% (NS) and 45% (*p* < 0.05), respectively, in the old subjects. HIF-1α protein expression was not modified by either age or treatment.

## 4. Discussion

The main finding of the present study is that the activation of the mTORC1 pathway by exercise and proteins is reduced in skeletal muscle of older subjects compared with young subjects. Our results indicate that the reduced activation of the mTORC1 pathway could be due to an increased expression of REDD1 and a reduced response to insulin. This is the first time that the protein expression of REDD1 is found to be upregulated in response to anabolic stimuli in human skeletal muscle of elderly people concomitantly to a reduction in the activation of the mTORC1 pathway.

This state of anabolic resistance is a controversial issue as some studies do not report any impairment in the response to anabolic stimuli in the elderly [18,19,35] while others do [4,9,12]. In the latter studies, impairments in protein digestion and amino acid absorption [36], insulin-mediated muscle tissue perfusion [37], amino acid uptake in muscle [38], or a reduced amount or activation status of key signaling proteins [4,11,12] have all been proposed to contribute to the anabolic resistance of muscle protein synthesis with aging. Here we specifically show that the response of PKB to resistance exercise coupled to the ingestion of a protein-rich drink is impaired in old subjects. Noteworthy the response of PKB to the protein-rich drink alone was not altered, which contrasts somewhat with the response of S6K1. The response to exercise combined with protein ingestion (*p* = 0.0652) as well as the response to protein ingestion alone (*p* = 0.0589) tended to be lower in the old group. The reduced phosphorylation state of PKB and S6K1 indicates that the response to resistance exercise and protein ingestion, two well-known anabolic stimuli in skeletal muscle, is impaired in our group of older subjects. The phosphorylation state of PKB and S6K1 after protein ingestion and resistance exercise was about 30% less in the old compared to the young group, which is in line with the response find by Kumar *et al.* [4] and between the highest value (−50% [11,12]) and the lowest value (−10% [39]) found in similar previous studies. Interestingly, basal PKB and S6K1 phosphorylation was not different between groups while a recent report on a relatively high number of subjects showed that basal mTORC1 signaling was higher in the elderly without modifying the protein synthesis rate [40]. Our results confirm the findings of a high number of previous studies reporting no difference in PKB and S6K1 phosphorylation at basal between young and older individuals [11,14,39,41], nor in muscle protein synthesis [4,11,14,39]. Due to its role at the intersection between PKB and mTORC1, the phosphorylation state of PRAS40 was analyzed. Contrary to the other components of the cascade, PRAS40 phosphorylation was higher after exercise and protein ingestion in the older group only, while we logically expected a lower phosphorylation state. We concluded that PKB is probably not the predominant kinase for the specific site Thr^246^ [42]. Although we did not measure its activity, PIM1 could be a possible candidate for the higher phosphorylation observed in the old subjects [43], which could represent a compensatory mechanism tending to increase mTORC1 activity. mTORC1 may be considered as a molecular hub receiving positive and negative inputs and integrating these signals. Even if PRAS40 phosphorylation state was higher in older subjects, the activity of mTORC1, assessed by the phosphorylation state of S6K1 was higher in the young group, likely because negative signals gained the upper hand. It is likely that changes in the phosphorylation of the regulatory-associated protein of mTOR (Raptor) [44,45] and/or dephosphorylation of DEP-domain-containing mTOR-interacting protein (Deptor) [46], two potential inhibitory signals on mTORC1 overcame the activating signal induced by PRAS40, globally resulting in a lower activation of mTORC1 in the elderly compared to the young group.

A global upregulation of REDD1 expression was found in the older group compared with the young group, possibly contributing to the decreased phosphorylation of PKB and S6K1 after resistance exercise and protein ingestion. To the best of our knowledge, this is the first study showing an increased REDD1 expression at the protein level in skeletal muscle of old subjects. This observation is in the same line as the differentiated regulation of REDD1 mRNA expression observed after resistance exercise in young and old women [17]. In young women, the mRNA level of REDD1 decreased after resistance exercise, thereby probably favoring the activation of the mTORC1 pathway, while in old women, no change was observed. The authors made the hypothesis that the lack of decrease in REDD1 in older women could contribute to the lower activation of the mTORC1 pathway after resistance exercise compared with younger women. Our findings re-enforce this hypothesis. However, it still remains to understand why REDD1 is more expressed in skeletal muscle in the elderly. REDD1 expression is known to be regulated by hypoxia and more specifically by HIF-1α [47]. However, HIF-1α protein expression was unchanged by any condition and the lower mRNA level measured in the fasted state in old compared with young subjects is contrary to the hypothesis tested, *i.e.*, activation of HIF-1α could contribute to the higher REDD1 expression in the elderly. As it regulates REDD1 expression in skeletal muscle of rats [48], it is likely that insulin contributed to the higher expression of REDD1 in the elderly, particularly in protein and protein + exercise conditions.

As mentioned above, reduced protein digestion and amino acid absorption may contribute to anabolic resistance [36]. When looking at plasma branched-chain amino acid concentrations, we found a lower concentration for isoleucine after protein ingestion and exercise. As isoleucine is known to active the mTORC1 pathway [49], the lower extracellular concentration measured after protein ingestion and exercise could have participated to the lower activation of the mTORC1 pathway. However, this interpretation has to be made with caution, as we did not measure the intramuscular concentration of isoleucine. Insulin is another factor that regulates the mTORC1 pathway in skeletal muscle and old people have been shown to be less responsive to hyperinsulinemia than young people [37]. In the present study, we also found that the anabolic response, *i.e.*, the activation of the mTORC1 pathway, was less important in older compared with young subjects despite a higher plasma insulin concentration after protein ingestion and exercise. Insulin levels increased in both groups after protein ingestion and exercise, which is not surprising due to the well-known insulinogenic effect of proteins and more particularly of leucine [50]. As already observed by others [35], the insulin and the glucose levels were higher in the old compared with the young group after protein ingestion and resistance exercise. The authors stated that this post-prandial hyperinsulinemia was probably a compensatory mechanism to facilitate glucose and amino acid handling. Here, in line with the previous hypothesis, the fasted glucose concentrations were already higher in the older subjects compared with younger subjects, indicating that those subjects had some impairment in the handling of blood glucose. We did not directly test whether our subjects were insulin resistant but in sight of our results it is likely that they are less sensitive to insulin than the young subjects studied. Despite higher insulin concentrations after exercise and protein ingestion, the mTORC1 pathway was less activated in the old than in the young group. Together with the higher expression of REDD1, it is likely that an impaired anabolic sensitivity contributed to the lower activation of the mTORC1 pathway after resistance exercise and amino acid ingestion in the older subjects as it was already observed in type 2 diabetic patients [51].

Looking further at the possible mechanisms previously found to repress mTORC1 activity, we did not observe any change in the phosphorylation state of AMPK Thr^172^ neither after protein ingestion, nor after exercise, suggesting that AMPK activity was not modified. Nevertheless, the increase in ACC phosphorylation at Ser^79^, a downstream target of AMPK [30], observed in both groups after exercise contradicted our first idea. This apparent discrepancy can be due to a rapid dephosphorylation of AMPK after exercise or an inaccurately assessed AMPK activity by its phosphorylation state. As ACC was not modified by protein ingestion alone, it is likely that the increase in phosphorylation was mainly due to resistance exercise. As no age difference was observed at the level of AMPK or ACC, we concluded that those two molecules did not contribute to the decreased responsiveness of the mTORC1 pathway to anabolic stimuli in the older subjects.

Anabolic resistance mainly affects protein synthesis [52], still, an increase in protein degradation can also contribute to the loss of muscle mass with aging. The activation of the ubiquitin-proteasome and the autophagy pathway was therefore assessed. No difference due to age was found in any marker studied. The only trend observed was a tendency to a higher level of LC3bII and thereby a higher level of LC3bII/I in the older group compared with the younger group in all conditions tested. Interpreting this result with caution, it is possible that a slight higher number of autophagosomes are synthetized in the elderly [53]. However, the whole autophagy flux does not seem to be different between the younger and older subjects as the level of p62, a marker for autophagosome degradation [53], was unaffected with age. We hereby confirm that aging affects markers for protein synthesis more than markers for protein degradation in response to anabolic stimuli. Independently of age, GABARAP mRNA level increased after exercise and not after protein ingestion. GABARAP is a key gene activated by autophagy itself and is part of the so-called transcriptional response to autophagy [53]. The higher GABARAP level observed after exercise and protein ingestion is therefore contradictory to the global tendency to reduced LC3bII/I ratio after both anabolic conditions in the present study and different from the results of Fry *et al.* [22]. In the latter study, the ratio LC3bII/I and the mRNA level of GABARAP decreased similarly after resistance exercise in young and old subjects. Muscle samples were taken at baseline, after 3, 6, and 24 h while we were interested in the early response to exercise and protein ingestion. The post-exercise muscle biopsy was therefore taken 30 min after the end of the exercise, which could have contributed to a different regulation of GABARAP. It is possible that the increased expression of GABARAP due to exercise persisted in the present study but that this effect disappeared during the later recovery period. The transcriptional response is secondary to the activation of autophagy itself and it is possible that a temporal delay occurred between the regulation of the LC3bII/I ratio and the mRNA level of GABARAP. The different nutritional state could also be a factor contributing to the opposite regulation of GABARAP in the two studies. However, as the subjects received protein immediately after exercise, *vs.* remained fasted in Fry *et al.* [22], the nutritional state in the present study should have favored a decreased expression of GABARAP instead. It seems therefore that the opposite results between both studies are due to temporal more than nutritional issues.

Strengths and limitations. For the first time, markers for both protein synthesis and protein degradation were systematically measured within the same study. One limitation is the lack of direct measurement for both processes, which implicates cautious interpretation of the data regarding protein synthesis and degradation, but not regarding signaling. Our sample of old subjects was comprised of six men and four women while it has previously been proposed that postmenopausal women may show more anabolic resistance in skeletal muscle than old men [54]. As no gender difference in the response to anabolic stimuli has been found as well [55] and as no difference was found between men and women on the different markers studied here, we are confident we could pool the data to increase the power of the study. Lastly, one pre and only one post sampling time point have probably limited the interpretation of the data. However, repeating muscle biopsies over time was not ethically acceptable, certainly in the older subjects.

In conclusion, this study confirms that aging affects markers for protein synthesis more than markers for protein degradation in response to anabolic stimuli. We show that the activation of the mTORC1 pathway is reduced in skeletal muscle of older subjects after resistance exercise and protein ingestion compared with young subjects. Mechanistically, our results suggest that REDD1 contributes to the anabolic resistance observed in the present study. If future research confirms this mechanism, REDD1 could constitute an interesting target to counteract sarcopenia.
